# Supplementary material for: Gene therapy delivery of anti-Müllerian hormone in prepubertal female domestic cats induces long-term sterilization
Source: Nat Commun. 2025 Nov 28;16:10747. doi: 10.1038/s41467-025-65780-2 (PMC12663202; doi:10.1038/s41467-025-65780-2)
Supplement: Supplementary file 1 — Supplementary Information [file 41467_2025_65780_MOESM1_ESM.pdf]

Supplementary Information

Gene therapy delivery of anti-Müllerian hormone in prepubertal female domestic cats induces long-term sterilization

Philippe Godin[1], Nicholas Nagykery[1], Natalie Sicher[1], Julie L. Barnes[2], Amy G. Miller[2], Christina Bunner[2], Amy K. Thompson[2], Motohiro Kano[1], Guangping Gao[3], Dan Wang[3], Patricia K. Donahoe[1], Linda Rhodes[4], David A. Brake[4], Thomas J. Conlon[4], William F. Swanson[2], Lindsey M. Vansandt[2] & David Pepin[1*]*

[1]Pediatric Surgical Research Laboratories, Massachusetts General Hospital, Department of Surgery, Harvard Medical School, Boston, United States.

[2]Center for Conservation and Research of Endangered Wildlife (CREW), Cincinnati Zoo and Botanical Garden, Cincinnati, United States.

[3]Horae Gene Therapy Center, University of Massachusetts Chan Medical School, Worcester, United States.

[4]Michelson Found Animals Foundation Inc., Los Angeles, United States.

*Co-corresponding authors. Emails: lindsey.vansandt@cincinnatizoo.org, dpepin@mgh.harvard.edu.

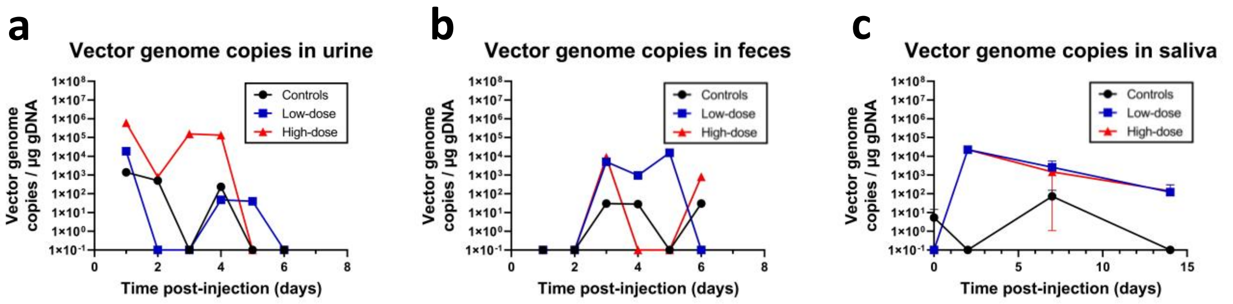

Supplementary Figure 1. Vector excretion in male and female domestic kittens injected with $5x10^{12}$ viral particles per kilogram of body weight of AAV9-Empty vector (Controls, black circles), or with $5x10^{12}$ (Low-dose, blue squares) or $1x10^{13}$ (High-dose, red triangles) viral genomes per kilogram of body weight of AAV9-fcMISv2. Vector genome quantification by qPCR in pooled urine (**a**) and fecal (**b**) samples collected during the first week post-injection, and in individual oral swab samples (**c**) collected weekly for two weeks. Samples with undetectable vector genome quantities were plotted as 0.1. Data in **c** are shown as means ± SD, *n* = 3, 5 and 4 cats in the control, low-dose and high-dose groups, respectively. Source data are provided as a Source Data file.

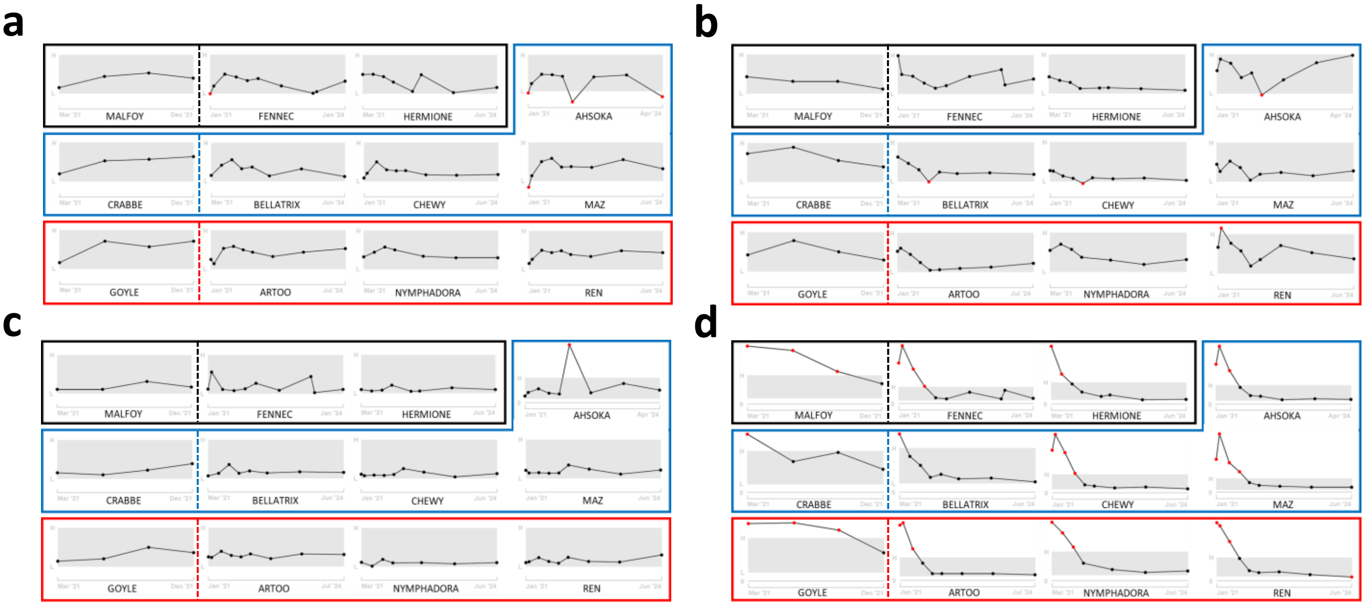

Supplementary Figure 2. Individual hematocrit (**a**), white blood cell (WBC) count (**b**), alanine aminotransferase (ALT) (**c**), and alkaline phosphatase (ALP) (**d**) profiles of domestic cats injected prepubertally with 5×10^{12} viral particles per kilogram of body weight of AAV9-Empty vector (Controls, black frame. *n* = one male, two females), or 5×10^{12} (Low-dose, blue frame. *n* = one male, four females) or 1×10^{13} (High-dose, red frame. *n* = one male, three females) viral genomes per kilogram of body weight of AAV9-fcMISv2. The leftmost cat of each group is a male. Blood samples were collected prior to study enrollment, and at three-, six-, nine-, twelve-, eighteen-, twenty-six-, and thirty-eight-months post-injection, with the twelve-month and subsequent timepoints only collected in females. Datapoints in red were outside the normal range (grey shading). Graphs were generated by the IDEXX VetConnect PLUS digital platform.

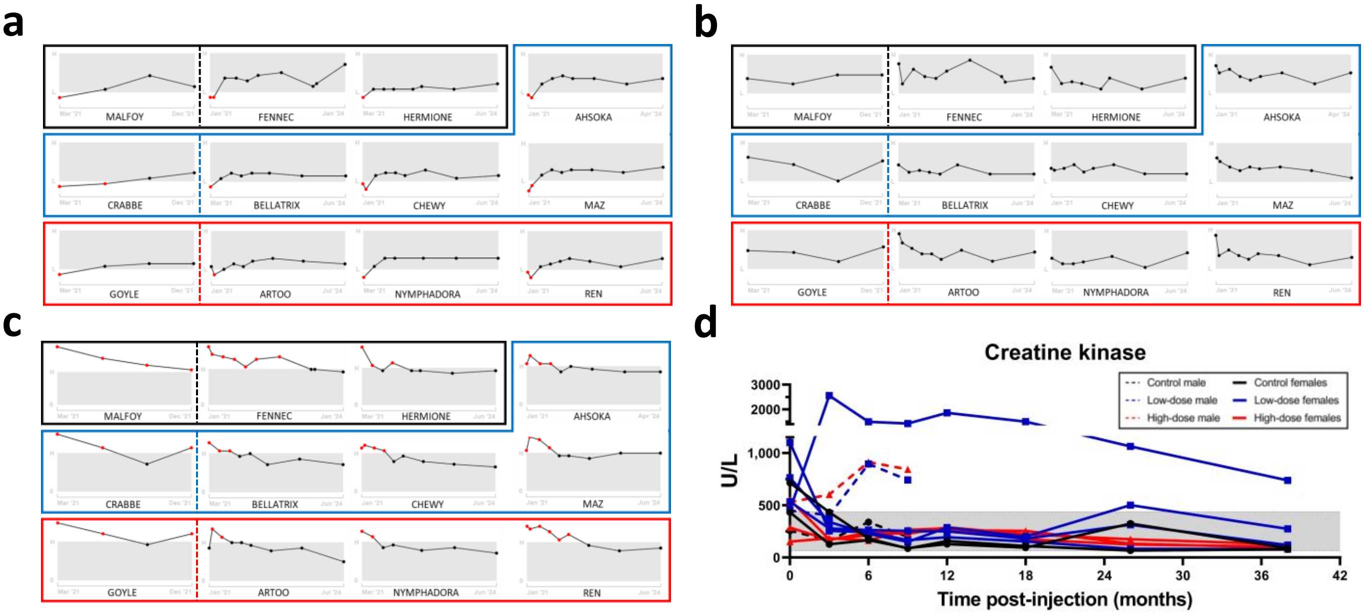

Supplementary Figure 3. Individual creatinine (**a**), blood urea nitrogen (BUN) (**b**), and symmetric dimethylarginine (SDMA) (**c**) profiles of domestic cats injected prepubertally with $5x10^{12}$ viral particles per kilogram of body weight of AAV9-Empty vector (Controls, black frame. *n* = one male, two females), or with $5x10^{12}$ (Low-dose, blue frame. *n* = one male, four females) or $1x10^{13}$ (High-dose, red frame. *n* = one male, three females) viral genomes per kilogram of body weight of AAV9-fcMISv2. The leftmost cat of each group is a male. Blood samples were collected prior to study enrollment, and at three-, six-, nine-, twelve-, eighteen-, twenty-six-, and thirty-eight-months post-injection, with the twelve-month and subsequent timepoints only collected in females. Datapoints in red were outside the normal range (grey shading). Graphs were generated by the IDEXX VetConnect PLUS digital platform. (**d**) Individual creatine kinase profiles of male (dotted lines) and female (solid lines) kittens of control (black), low- (blue), and high-dose (red) groups during the same period. Source data are provided as a Source Data file.

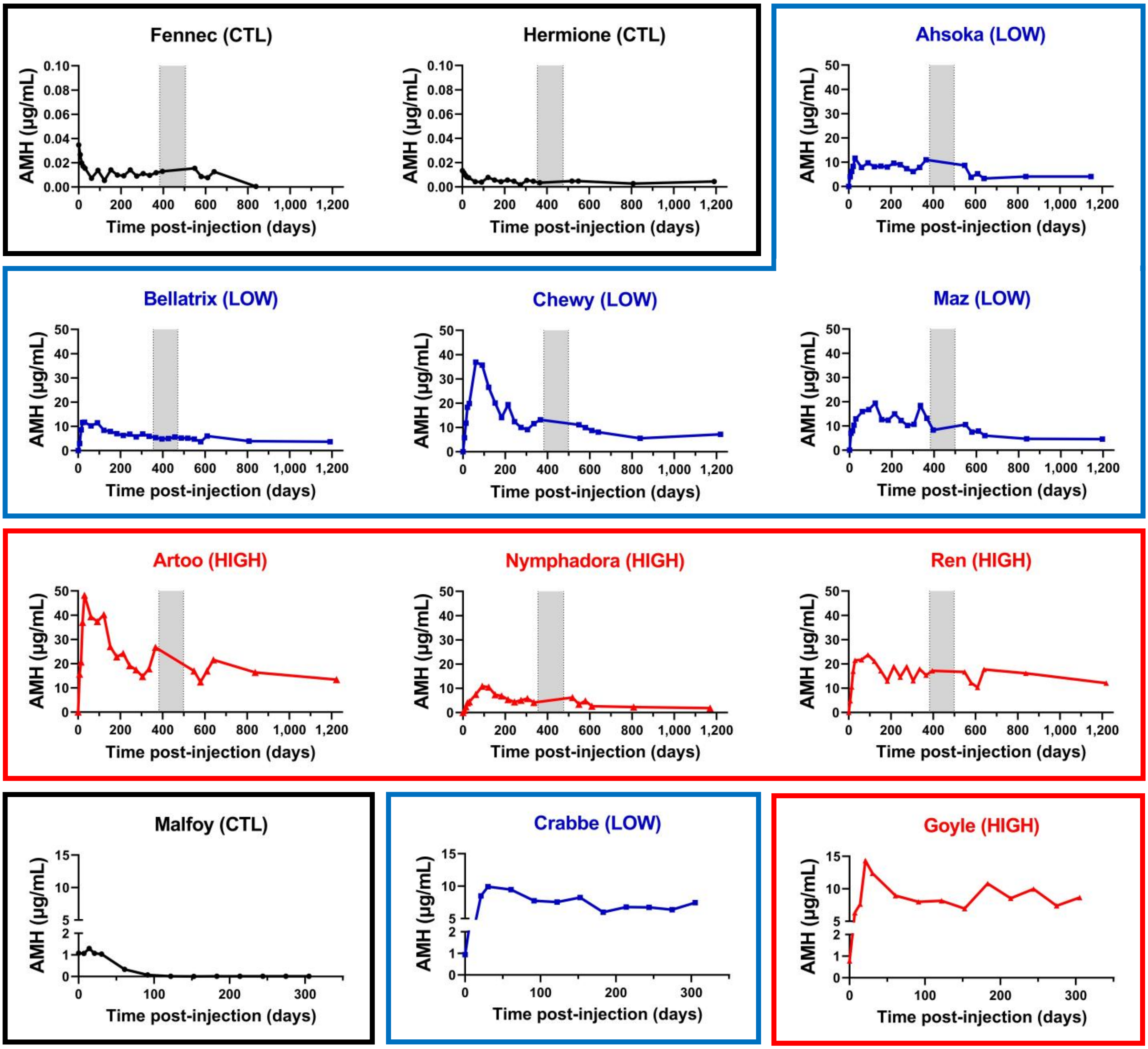

Supplementary Figure 4. Individual serum AMH profiles in female (first three rows) and male (fourth row) domestic kittens injected with $5x10^{12}$ viral particles per kilogram of body weight of AAV9-Empty vector (Controls, black), or with $5x10^{12}$ (Low-dose, blue) or $1x10^{13}$ (High-dose, red) viral genomes per kilogram of body weight of AAV9-fcMISv2. Note that the y-axis scale range differs between control (0.0-0.1) and treated (0-50) females. Mating trial periods are shaded in grey in female profiles. Source data are provided as a Source Data file.

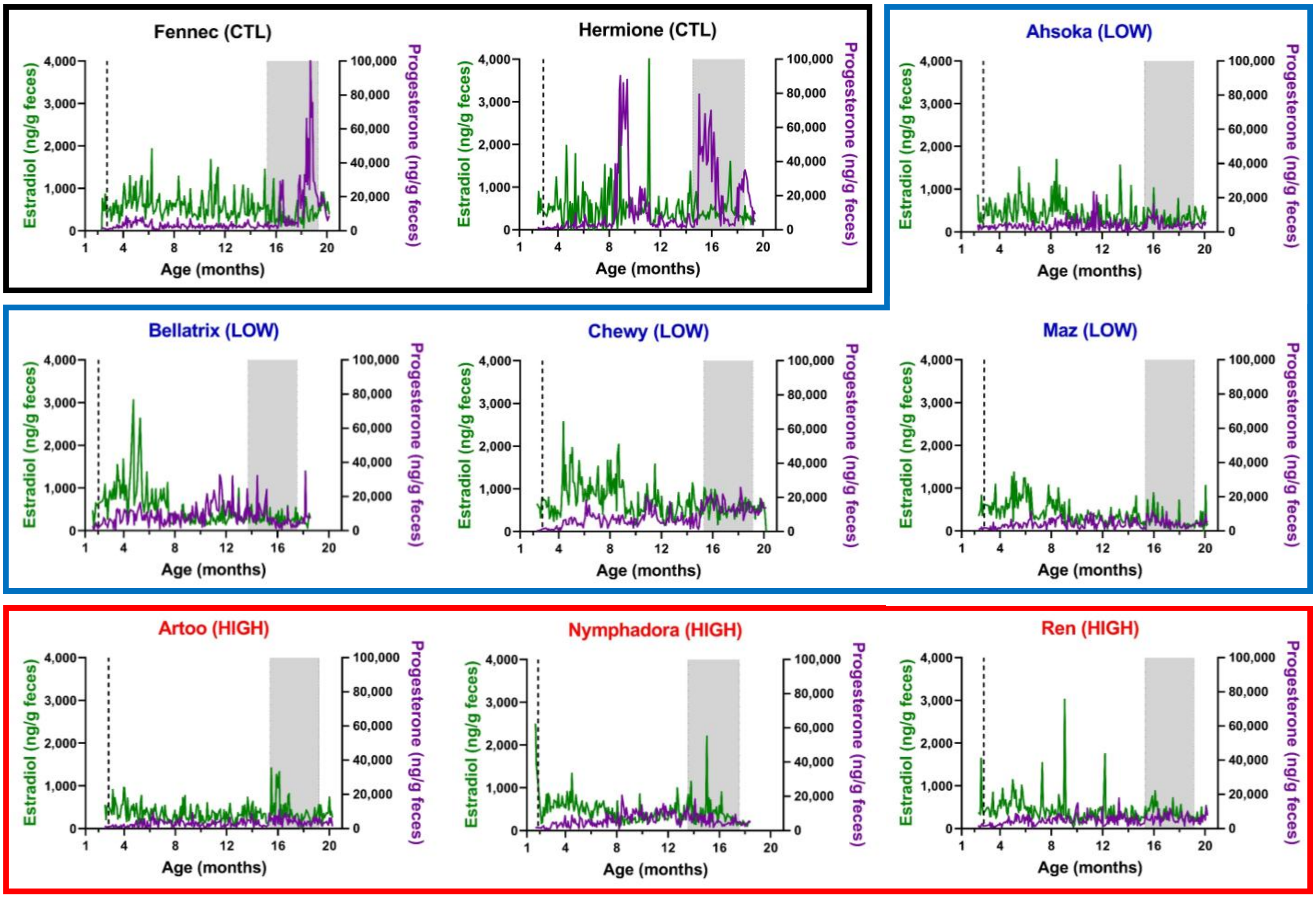

Supplementary Figure 5. Individual fecal estrogen (green) and progestogen (purple) metabolite levels in female domestic cats injected prepubertally with $5x10^{12}$ viral particles per kilogram of body weight of AAV9-Empty vector (Controls, black), or with $5x10^{12}$ (Low-dose, blue) or $1x10^{13}$ (High-dose, red) viral genomes per kilogram of body weight of AAV9-fcMISv2. Vertical dashed lines indicate the day of injection while grey shaded regions indicate the mating trial period for every female. Source data are provided as a Source Data file.

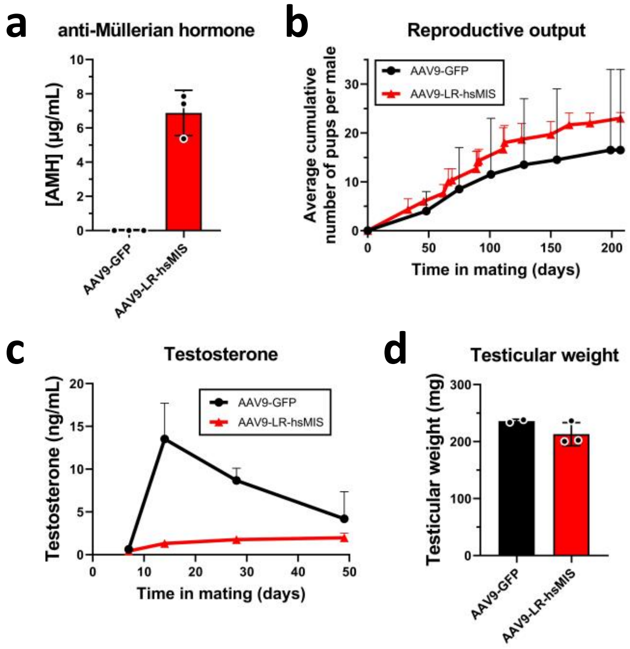

Supplementary Figure 6. Supraphysiological concentrations of AMH in male mice do not impact fertility. (**a**) Circulating AMH concentrations in adult male mice that received a single intraperitoneal injection of 3×10^{11} viral genomes per mouse of AAV9 carrying either a GFP transgene (AAV9-GFP, *n* = 2, black, circles) or a human AMH transgene (AAV9-LR-hsMIS, *n* = 3, red, triangles). (**b**) Average cumulative number of pups per male during a seven-month-long mating study. (**c**) Average circulating testosterone concentrations in both groups during the first two months of the study. (**d**) Average testicular weight at endpoint, seven months after the injection. Data are shown as means ± SEM. Source data are provided as a Source Data file.

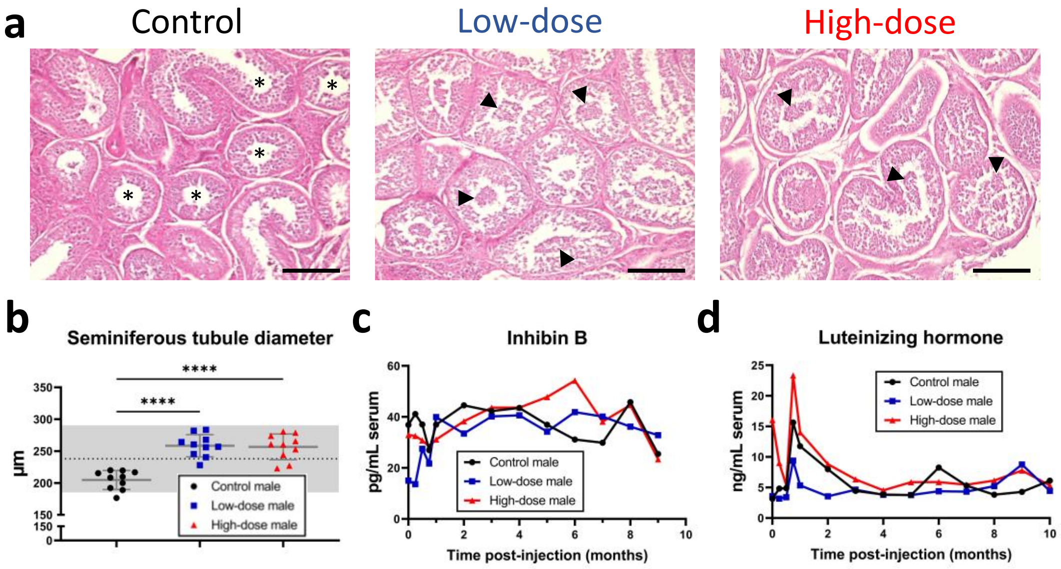

Supplementary Figure 7. (**a**) Representative histological sections of testes of male domestic cats injected prepubertally with $5x10^{12}$ viral particles per kilogram of body weight of AAV9-Empty vector (Control, black), or $5x10^{12}$ (Low-dose, blue) or $1x10^{13}$ (High-dose, red) viral genomes per kilogram of body weight of AAV9-fcMISv2 (*n* = one male per group). Orchiectomy was performed ten months after injection, when males were one year of age. Asterisks indicate the lumen of seminiferous tubule sections completely devoid of mature sperm in the control male. Arrowheads indicate mature sperm in the lumen of some seminiferous tubules in treated males. Scale bars are 200 µm. (**b**) The longer and shorter diameters of ten randomly-selected round seminiferous tubule sections were measured in each male. The average diameter for both the low-dose (blue squares) and the high-dose (red triangles) males was plotted and compared to the average seminiferous tubule diameter of the control male (black circles) using a one-way ANOVA (Dunnett's post hoc test): ****$P<0.0001$ for both comparisons. Data are shown as means ± SD. The horizontal dotted line and shaded region represent the average seminiferous tubule diameter ± SD (237.9 ± 52.5 µm) previously reported in sexually mature male domestic cats over six months of age[33]. Inhibin B (**c**) and luteinizing hormone (**d**) profiles in the serum of the male domestic cats throughout the study. Source data are provided as a Source Data file.

Supplementary Table 1. Characteristics of the study cats, group assignments, and treatment and mating trial details.

			Treatment administration				Mating trial	
	Sex	Date of birth	Treatment group[1]	Date of injection (age in days)	Weight at injection (kg)	Volume injected (ml)	Mating trial start date (age in days)	Breeding group
Malfoy	M	2021-01-13	Control	2021-03-24 (70)	1.26	0.65	N/A	N/A
Crabbe	M	2021-01-13	Low-dose	2021-03-24 (70)	1.17	0.55	N/A	N/A
Goyle	M	2021-01-13	High-dose	2021-03-24 (70)	1.17	1.00	N/A	N/A
Fennec	F	2020-12-03	Control	2021-02-24 (83)	1.28	0.66	2022-03-14 (466)	Bernie
Hermione	F	2020-12-27	Control	2021-03-24 (87)	1.73	0.85	2022-03-14 (442)	Tom
Ahsoka	F	2020-12-03	Low-dose	2021-02-24 (83)	1.30	0.55	2022-03-14 (466)	Bernie
Bellatrix	F	2021-01-21	Low-dose	2021-03-24 (62)	1.17	0.51	2022-03-14 (417)	Tom
Chewy	F	2020-12-03	Low-dose	2021-02-24 (83)	1.35	0.58	2022-03-14 (466)	Bernie
Maz	F	2020-12-02	Low-dose	2021-02-24 (84)	1.21	0.56	2022-03-14 (467)	Bernie
Artoo	F	2020-12-01	High-dose	2021-02-24 (85)	1.60	1.37	2022-03-14 (468)	Bernie
Nymphadora	F	2021-01-26	High-dose	2021-03-24 (57)	1.07	0.88	2022-03-14 (412)	Tom
Ren	F	2020-12-03	High-dose	2021-02-24 (83)	1.45	1.22	2022-03-14 (466)	Bernie

[1]Control cats received a single intramuscular injection of $5x10^{12}$ viral particles per kilogram of body weight of AAV9-Empty vector. Treated cats received a single intramuscular injection of either $5x10^{12}$ (Low-dose) or $1x10^{13}$ (High-dose) viral genomes per kilogram of body weight of AAV9-fcMISv2.

Supplementary Table 2. Secondary sexual characteristics, sperm parameters, and sperm fertilization capacity assessed during four complete reproductive evaluations of male domestic cats involved in the study and taken from reference data in the literature.

			Reproductive evaluation				Sperm motility & morphology			Sperm function			
Treatment group[1]	Semen collection method	Age (months)	Penile morphology	Semen volume (mL)	Total sperm count ($x10^6$)	Sperm concentration ($x10^6$/mL)	Sperm progressive motility (%)	Rate of progressive motility (0-5)	Normal sperm morphology (%)	Sperm motility (% progressive/rate)		Oocyte cleavage (%)	Fertilization (%)
										0 hpi	48 hpi		
Control	EEJ	5	Complete prepuce + Distinct spines	0.21	N/A	Aspermic	N/A	N/A	N/A				
	EEJ	8		0.33	0.0012	0.0035	N/A	N/A	N/A				
	EEJ	11		0.36	0.0074	0.0209	N/A	N/A	3	N/A	N/A	N/A	N/A
	Gamete rescue	12		N/A	0.117	N/A	N/A	N/A	N/A				
Low-dose	EEJ	5	Complete prepuce + Distinct spines	0.13	N/A	Aspermic	N/A	N/A	N/A				
	EEJ	8		0.10	7.35	75	70	3.5	20				
	EEJ	11		0.18	26.91	153	80	3.5	46	90/4.5	40/2.5	49 (18/37)	49 (17/35)
	Gamete rescue	12		N/A	32.17	N/A	60	3.5	N/A				
High-dose	EEJ	5	Penile frenulum + Distinct spines	0.11	N/A	Aspermic	N/A	N/A	N/A				
	EEJ	8	Complete prepuce + Distinct spines	0.21	55.23	263	80	4.0	24				
	EEJ	11		0.21	79.19	384	80	4.0	49	80/4.0	50/2.5	36 (13/36)	31 (11/35)
	Gamete rescue	12		N/A	22.01	N/A	70	3.5	N/A				
Reference data from the literature	Zambelli *et al.*, 2008 [35]			0.07 ± 0.03		543 ± 578	78 ± 10		92 ± 2				45
	Filliers *et al.*, 2010 [36]			--		--	50 ± 20		42 ± 19				40 ± 2
	Nuñez Favre *et al.*, 2012 [37]			0.18 ± 0.01		172 ± 17	87 ± 2		64 ± 1				--

[1]Control cats received a single intramuscular injection of $5x10^{12}$ viral particles per kilogram of body weight of AAV9-Empty vector. Treated cats received a single intramuscular injection of either $5x10^{12}$ (Low-dose) or $1x10^{13}$ (High-dose) viral genomes per kilogram of body weight of AAV9-fcMISv2.

EEJ = Electroejaculation. hpi = hours post-insemination.
