# Supplementary material for: Gene therapy delivery of anti-Müllerian hormone in prepubertal female domestic cats induces long-term sterilization
Source: Nat Commun. 2025 Nov 28;16:10747. doi: 10.1038/s41467-025-65780-2 (PMC12663202; doi:10.1038/s41467-025-65780-2)
Supplement: Supplementary file 2 — Reporting Summary [file 41467_2025_65780_MOESM2_ESM.pdf]

nature portfolio

Corresponding author(s): Lindsey M. Vansandt & David Pepin

Last updated by author(s): Sep 29, 2025

Reporting Summary

Nature Portfolio wishes to improve the reproducibility of the work that we publish. This form provides structure for consistency and transparency in reporting. For further information on Nature Portfolio policies, see our Editorial Policies and the Editorial Policy Checklist.

Statistics

For all statistical analyses, confirm that the following items are present in the figure legend, table legend, main text, or Methods section.

n/a	Confirmed	
☐	☒	The exact sample size (*n*) for each experimental group/condition, given as a discrete number and unit of measurement
☐	☒	A statement on whether measurements were taken from distinct samples or whether the same sample was measured repeatedly
☐	☒	The statistical test(s) used AND whether they are one- or two-sided<br>*Only common tests should be described solely by name; describe more complex techniques in the Methods section.*
☒	☐	A description of all covariates tested
☒	☐	A description of any assumptions or corrections, such as tests of normality and adjustment for multiple comparisons
☐	☒	A full description of the statistical parameters including central tendency (e.g. means) or other basic estimates (e.g. regression coefficient) AND variation (e.g. standard deviation) or associated estimates of uncertainty (e.g. confidence intervals)
☐	☒	For null hypothesis testing, the test statistic (e.g. *F*, *t*, *r*) with confidence intervals, effect sizes, degrees of freedom and *P* value noted<br>*Give P values as exact values whenever suitable.*
☒	☐	For Bayesian analysis, information on the choice of priors and Markov chain Monte Carlo settings
☒	☐	For hierarchical and complex designs, identification of the appropriate level for tests and full reporting of outcomes
☒	☐	Estimates of effect sizes (e.g. Cohen's *d*, Pearson's *r*), indicating how they were calculated

Our web collection on statistics for biologists contains articles on many of the points above.

Software and code

Policy information about availability of computer code

Data collection	Real-time PCR data were collected using the QuantStudio 3 real-time PCR system (Thermo Fisher Scientific).<br>Luminescence was detected using the Wallac 1420 Victor2 microplate reader (PerkinElmer) and the Wallac 1420 version 3.00 software (PerkinElmer).<br>The seminiferous tubule diameters were measured using ImageJ version 1.53m.
Data analysis	Real-time PCR data were analyzed using the QuantStudio Design & Analysis version 1.4.1 software (Thermo Fisher Scientific).<br>Quantitative analysis of ELISA data was performed using the “Four Parameter Logistic Curve” online data analysis tool (MyAssays Ltd.) accessible at https://www.myassays.com/four-parameter-logistic-curve.assay.<br>Progestogen metabolite baseline values were calculated using the R statistical package hormLong (version 1.0).<br>All other data analyses and graphing were performed using GraphPad Prism version 9.3.1.

For manuscripts utilizing custom algorithms or software that are central to the research but not yet described in published literature, software must be made available to editors and reviewers. We strongly encourage code deposition in a community repository (e.g. GitHub). See the Nature Portfolio guidelines for submitting code & software for further information.

Data

Policy information about availability of data

All manuscripts must include a data availability statement. This statement should provide the following information, where applicable:

- Accession codes, unique identifiers, or web links for publicly available datasets
- A description of any restrictions on data availability
- For clinical datasets or third party data, please ensure that the statement adheres to our policy

The domestic cat (Felis silvestris catus) anti-Müllerian hormone (AMH) NCBI reference sequence mentioned in this work is XP_011286375.2 and can be found at https://www.ncbi.nlm.nih.gov/protein/XP_011286375.2. All data supporting the findings of this study are available within the paper and its Supplementary Information and Source Data files. A reporting summary is available as Supplementary Information file.

Human research participants

Policy information about studies involving human research participants and Sex and Gender in Research.

Reporting on sex and gender	N/A
Population characteristics	N/A
Recruitment	N/A
Ethics oversight	N/A

Note that full information on the approval of the study protocol must also be provided in the manuscript.

Field-specific reporting

Please select the one below that is the best fit for your research. If you are not sure, read the appropriate sections before making your selection.

☒ Life sciences ☐ Behavioural & social sciences ☐ Ecological, evolutionary & environmental sciences

For a reference copy of the document with all sections, see nature.com/documents/nr-reporting-summary-flat.pdf

Life sciences study design

All studies must disclose on these points even when the disclosure is negative.

Sample size	The main comparison in this study was the reproductive output of control AAV9-empty female cats versus treatment with AAV9-fcMISv2. Our previous published study (Vansandt et al., 2023; DOI: 10.1038/s41467-023-38721-0) that assessed the reproductive output of adult females after the same treatment used three animals per group. It successfully showed that elevated AMH serum levels resulted in complete infertility of treated females. In the current study, we elected for groups of n=2 for controls to limit unnecessary births.<br>The impact of the AAV9-LR-hsMIS treatment on male fertility in mice was evaluated in n=3 treated animals. Since complete infertility had to be attained to justify the use of such sterilant in free-roaming males, it was deemed sufficient to detect such a drastic difference.
Data exclusions	Apart from the low-dose cat that did not mate (Bellatrix) being removed for comparison of estrous behavior between controls and treated females, no data were excluded from the analyses.
Replication	Female cats were treated with two dose groups to ensure reproducibility.<br>ELISAs were run with technical replicates.<br>Target vgc (vector genome copies) present in gDNA were assayed in triplicate by real time PCR.<br>All attempts at replication were successful.<br>For fecal estrogens, androgens and progestogens: only one technical replicate per timepoint was run.
Randomization	Animals were randomly assigned to experimental groups using the random number generator function in Microsoft Excel.
Blinding	Investigators were not blinded to the treatment group allocation of the cats. The authors who administered the treatment to the cats were also the veterinarians in charge of data collection. However, male-female interactions during mating trial were scored by blinded investigators.<br>The investigator scoring the seminiferous tubule diameters in male cats was blinded to the treatment.

Reporting for specific materials, systems and methods

We require information from authors about some types of materials, experimental systems and methods used in many studies. Here, indicate whether each material, system or method listed is relevant to your study. If you are not sure if a list item applies to your research, read the appropriate section before selecting a response.

Materials & experimental systems

n/a	Involved in the study
☐	☒ Antibodies
☒	☐ Eukaryotic cell lines
☒	☐ Palaeontology and archaeology
☐	☒ Animals and other organisms
☒	☐ Clinical data
☒	☐ Dual use research of concern

Methods

n/a	Involved in the study
☒	☐ ChIP-seq
☒	☐ Flow cytometry
☒	☐ MRI-based neuroimaging

Antibodies

Antibodies used	1. Polyclonal goat anti-feline IgG (H+L) HRP (Novus Biologicals, catalog # NBP1-73347, lot # 4168).<br>2. Monoclonal mouse anti-human recombinant AMH antibody (produced in-house, clone 6E11).<br>3. Polyclonal rabbitl anti-AMH antibody (produced in-house, clone MGH6).<br>4. Polyclonal donkey anti-rabbit IgG HRP (Jackson ImmunoResearch Laboratories, catalog # 711-035-152).<br>5. Polyclonal anti-17β-estradiol (supplied by Coralie J. Munro – University of California, clone R4972).<br>6. Monoclonal mouse anti-bovine LHβ antibody (supplied by Janet F. Roser – University of California, clone 518-B7).
Validation	1. The anti-AMH antibody ELISA was validated by using serum samples of a cat that developed anti-AMH antibodies following the administration of a previous version of the gene therapy as positive controls. The manufacturer states that it works for feline samples and for ELISA applications. We used the same antibody as in Vansandt, L. M. et al. Durable contraception in the female domestic cat using viral-vectored delivery of a feline anti-Müllerian hormone transgene. Nat. Commun. 14, 3140 (2023).<br>2, 3 and 4. The measurement of human AMH in the mouse serum samples using these antibodies is validated by the close-to-undetectable levels of AMH in mice receiving empty-AAV9 particles and with the supraphysiological levels of AMH detected in mice injected with AAV9-LR-hsMIS. It was performed as previously reported in Pepin, D. et al. An albumin leader sequence coupled with a cleavage site modification enhances the yield of recombinant C-terminal Mullerian Inhibiting Substance. Technology 1, 63-71 (2013).<br>5. The same antibody and procedure was used in Vansandt, L. M. et al. Durable contraception in the female domestic cat using viral-vectored delivery of a feline anti-Müllerian hormone transgene. Nat. Commun. 14, 3140 (2023). This antibody has been validated for other feline (and non-feline) species (see references included in the Methods section). 17β-estradiol, as a steroid hormone, has the same structure for every species.<br>6. LH concentrations were analyzed by the Wildlife Endocrinology Research Laboratory (Smithsonian's National Zoo & Conservation Biology Institute).The assay was validated for cat serum through tests of parallelism, accuracy (spike-and-recovery), and sensitivity. The same antibody and procedure was used in Vansandt, L. M. et al. Durable contraception in the female domestic cat using viral-vectored delivery of a feline anti-Müllerian hormone transgene. Nat. Commun. 14, 3140 (2023).

Animals and other research organisms

Policy information about studies involving animals; ARRIVE guidelines recommended for reporting animal research, and Sex and Gender in Research

Laboratory animals	- Mouse experiments were conducted with eight-weeks-old C57BL/6 male mice (Charles River Laboratories). They were housed in 12 hours light/12 hours night conditions with room temperature and humidity maintained between 20-23 °C and 30-70%, respectively. Mice had unlimited access to Prolab® IsoPro® RMH 3000 (LabDiet, catalog # 5P76) rodent chow and water.<br><br>- Cat experiments were performed using domestic cats (Felis silvestris catus) maintained in a research colony at the Cincinnati Zoo and Botanical Garden’s Center for Conservation and Research of Endangered Wildlife (CREW). Female and male cats were 2.0-3.0 months old at treatment (see Supplementary Table 1 for individual ages). Six female kittens were purchased commercially (Marshall BioResources), while the other kittens were born at the CREW facility. They were housed under a 14:10 h light:dark cycle, fed Purina Pro Plan Development Chicken & Rice kitten dry food (Nestle Purina Petcare), and provided access to fresh water ad libitum throughout the study. Food was switched to Purina Pro Plan Complete Essentials Chicken & Rice adult dry ration (Nestle Purina Petcare) when study animals were approximately one year of age. To facilitate fecal sample identification by individual cat, cats were fed small amounts of Purina Pro Plan Development Chicken & Liver or Complete Essentials Beef & Carrots Entrée kitten and adult wet food (Nestlé Purina PetCare) containing food-grade dye (Wilton Brands) and/or glitter (Dixon Ticonderoga Company) on the night preceding sample collection. Kittens were group housed under Biosafety Level 2 containment according to their treatment group for five days after vector administration and then returned to a single colony room for group housing. Male kittens were moved to individual enclosures two months after treatment administration to prevent potential breeding and fighting, while females were group-housed in a single room until the mating trial. During the mating trial, females were separated in two different rooms. The two proven male breeders (2.3 and 2.8 years old at the initiation of the mating trial) were singly housed outside of breeding periods.
Wild animals	No wild animals were used in this study
Reporting on sex	The sex of the animals is clearly reported for every experiment included in this study. All data is reported disaggregated for sex.
Field-collected samples	No field collected samples were used in this study

Ethics oversight	Experiments in mice were performed in accordance with the experimental protocol 2014N000275 approved by the Massachusetts General Hospital Institutional Animal Care and Use Committee.<br>All cat procedures were approved by the Cincinnati Zoo and Botanical Garden's Institutional Animal Care and Use Committee (Identification Number 22–170) and the Cincinnati Children's Hospital Medical Center Institutional Biosafety Committee (IBC 2021-0071).

Note that full information on the approval of the study protocol must also be provided in the manuscript.
